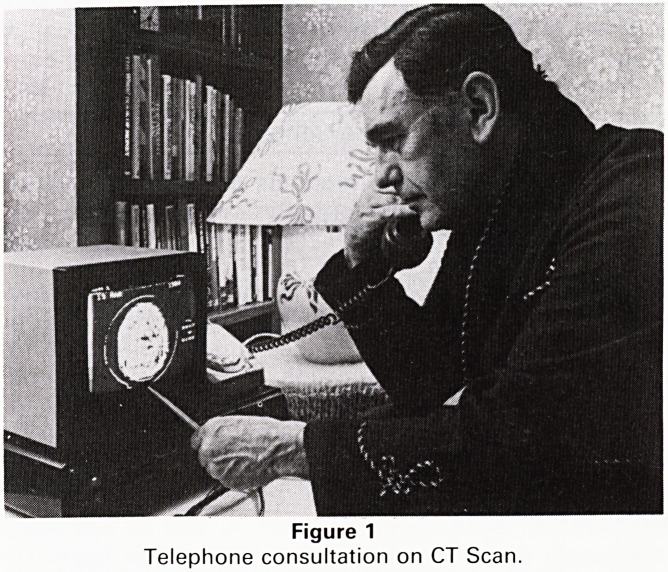# The Imtran Revolution, Telephone Transmission of X-Rays and Scans


**Published:** 1986-04

**Authors:** Huw Griffith

**Affiliations:** Consultant Neurosurgeon, Frenchay Hospital, Bristol


### The Imtran* Revolution—Telephone Transmission of X-rays and Scans

**Huw Griffith**
Consultant Neurosurgeon, Frenchay Hospital, Bristol

Eleven years ago the CT scanner was introduced into medicine world-wide. The pictures were generated by video or digital type information (photographed on to paper or film) put together to make an anatomical picture. It was not of course simply for this aspect alone that it made its impact. We already had the isotope linear scaner and gamma camera generating pictures some of which were video outputs with digital information. The presentation of the material in a simple anatomical slice made it possible for surgeons to interpret the pictures without difficulty and to act directly as a result of making the diagnosis, particularly in emergencies. At first the CT scanners were confined to neurosurgical units but they slowly spread outwards to other centres. In this country there are now approximately 120 CT scanners and only about 30 or so neurosurgical centres. The situation arose in this region where scanners elsewhere were generating pictures of great interest to a neurosurgeon who was not actually on the spot. The only way in which the surgeon could know what was in the picture was to listen to a verbal description on the telephone, often by a non-expert, or to have the scans brought or sent by general post or courier. Clearly this was not going to be satisfactory. With the Physics Department at Frenchay, I instituted a study in 1978 to see whether we could send the pictures down telephone lines or along carrier waves so that we could give better advice, and arrange more expeditious and well directed transfers and treatment, in short, to give a better service than we could without the pictures. At that stage it looked as if it would not be possible economically so to do although you will recall that the world had already watched on television, pictures transmitted from moon to earth.

However in 1981 with the local British Telecom we tried to initiate a pilot study using the simpler gamma camera pictures to see whether we could work towards a telephone transmission for CT pictures. This came to nothing. It was not until the end of 1983 I managed to generate sufficient steam behind this project to get British Telecom really interested in it. By that time their research branch at Martlesham had developed a security surveillance system by which up to four video cameras, possibly mounted in warehouses or shops, could be monitored by telephone from one central location. With some adaptations it looked as if we could use it to transmit CT pictures down telephone lines. So it proved. We saw that the picture quality was virtually the same as looking at the video screen on the scanner. This was clearly going to be a major force in neurosurgical management. What we did not then realise was how it was going to influence other branches of medicine and medicine as a whole.

Those who have seen the present system working are impressed by its simplicity. At home I have a box, Fig. 1 approximately 3½″ thick×11″ deep×15″ wide. It is next to one of my telephones which has an ordinary jack plug socket. On top sits a black and white television monitor with a 10″ screen costing about £120. Operation is simple. Either by ringing up the hospital or by the hospital

**Figure 1**
Telephone consultation on CT Scan.

ringing up, the connection is made. A picture is 'captured' into the transmission box on the scanner. This is identical with the receiving box so that pictures can be exchanged from either end. Simply by pressing another button, the image is transmitted. There are three different resolutions of transmission, respectively on low, normal and high resolution, at 8, 30 and 60 seconds respectively. CT pictures ordinarily take 30 seconds. A chest x-ray is best transmitted at 60 seconds. At first we were worried lest there be some loss of detail in the chest x-ray transmission but this has been very easily solved by putting a zoom lens on the black and white video camera necessary to transmit the chest x-ray picture. This enables the detail transmitted to exceed that visible to the naked eye simply by zooming up on the area in question. Now instead of an entire chest x-ray you are transmitting a magnified part of one.

In a series of head scans there will be six or eight pictures to see. One method of proceeding is to view them all by flipping quickly through the set at 8 seconds each and then having a more leisurely examination of the two or three crucial ones with greater resolution. Usually one is dealing at the other end of the line with another doctor so that immediately the picture is transmitted you flip back over into 'talk mode', when the picture or clinical details can be discussed.

What we are really doing is carrying out a true consultation just as if we were in the ward or x-ray department or clinic with the x-rays in front of us. It is clear that this will sharpen and speed up and perhaps make more accurate an enormous range of these consultations where two doctors communicate with one another. It is easy to see how this could work in hospital practice, particularly between hospitals. If a hospital doctor is referring a patient to another hospital for treatment then it has in the past been necessary for a letter to be typed out with the details of the case and if the x-rays are relevant, as they usually are, for those pictures to be put into a large envelope and sent through the post which has really remained much as it was in 1852. The envelopes then have to be transmitted, sorted and delivered to the right department before the next stage can

*(Continued on page 37)*

* Newspeak for Image Transmission.

### The Imtran Revolution *(continued from page 29)*

begin. The Imtram system cuts through all that and moreover, because the clinicians are talking to one another, any little details that could delay matters can be ironed out there and then. The result should be a better service to patients. In addition, because doctors will be talking directly to one another there will be an educational spin-off since as the films are discussed together with the clinical details, the one who knows less will learn more as this person-to-person type of consultation gives the opportunity for asking and answering questions.

There are still more doctors in general practice than in any other specialty. How is this going to affect them? It depends on how much they want it to. At present if a general practitioner requests a chest x-ray, the patient travels to the hospital or clinic and has the x-ray. What the general practitioner gets in return is the radiologist's written opinion. With the Imtran system in his surgery he will be able to get the chest x-ray transmitted to view directly just as he did when he was a hospital doctor. Moreover, he will be able to question the radiologist about the significance of details and to get advice on possible repeat films, referral and how the next move in the investigation and treatment of the patient might be made. This will inevitably involve the general practitioners much more in the interpretation of x-rays, their further patient management being guided by this information. However, how far this happens will of course depend on the practitioner. If he wishes to manage pneumonias at home with this device it will inevitably entail more responsibility. The financial and adminstrative structure of general practice at the moment does not encourage him to take on either the financing of the new device nor its useful deployment. Until this occurs the revolutionary impact of the new device may not be felt in general practice as much as it undoubtedly will in hospital medicine.

If retrieval of CT and x-ray images and the ability to send them down telephone lines is going to become a much larger feature of medicine, the storage of this radiological material will have to alter. We already know that sorting through packs of celluloid film in progressively more tattered envelopes is a real chore. Every time an image is translated into another mode it will be ever so slightly degraded so that rephotographing celluloid film is never going to be quite as good as the original. It therefore is better to store the images directly as digital pictures so that they can be accessed and retrieved by telephone. We are now feeling the impact of this in a regional unit when CT scanners are coming into commission in four other centres in the region and soon no doubt, more. Nuclear magnetic resonance (MR) scanners will now put out digitally based pictures which are better stored in data banks than on celluloid film in brown paper packets. So we shall have to develop large telephone line access storage devices in hospitals. This is anyway inevitable since the 'silvermine' of celluloid x-ray films needs more space and more labour than most hospitals are prepared to give it. The Imtran system will accelerate the need for this and also the need to generate x-ray films on devices other than on celluloid film.

In the South West of England we have started a medical revolution which will 'ring round the world'. It remains to be seen whether the world will make good use of this and in particular, whether British medical industry will exploit and benefit from yet another revolutionary British medical invention.

## Figures and Tables

**Figure 1 f1:**